# The arsenic bioremediation using genetically engineered microbial strains on aquatic environments: An updated overview

**DOI:** 10.1016/j.heliyon.2024.e36314

**Published:** 2024-08-22

**Authors:** Mohammed A.E. Naiel, Ehab S. Taher, Fatema Rashed, Shakira Ghazanfar, Abdelrazeq M. Shehata, Nourelhuda A. Mohammed, Raul Pascalau, Laura Smuleac, Ateya Megahed Ibrahim, Ahmed Abdeen, Mustafa Shukry

**Affiliations:** a Animal Production Department, Faculty of Agriculture, Zagazig University, Zagazig, 44519, Egypt; b Department of Basic Medical and Dental Sciences, Faculty of Dentistry, Zarqa University, Zarqa, 13110, Jordan; c National Institute for Genomics Advanced Biotechnology, National Agricultural Research Centre, Park Road, Islamabad, 45500, Pakistan; d Department of Animal Production, Faculty of Agriculture, Al-Azhar University, Cairo, Egypt; e Department of Physiology and Biochemistry, Faculty of Medicine, Mutah University, Mutah, 61710, Al-Karak, Jordan; f Department of Agricultural Technologies, Faculty of Agriculture, University of Life Sciences "King Mihai I" from Timisoara, Romania; g Department of Sustainable Development and Environmental Engineering Faculty of Agriculture, University of Life Sciences "King Mihai I" from Timisoara, Timisoara, Roman, Romania; h Department of Administration and Nursing Education, College of Nursing, Prince Sattam bin Abdulaziz University, Al-Kharj, 11942, Saudi Arabia; i Department of Family and Community Health Nursing, Faculty of Nursing, Port-Said University, Egypt; j Department of Forensic Medicine and Toxicology, Faculty of Veterinary Medicine, Benha University, Toukh, 13736, Egypt; k Department of Physiology, Faculty of Veterinary Medicine, Kafrelsheikh University, Kafrelsheikh, 33516, Egypt; l Department of Biochemistry, Faculty of Veterinary Medicine, South Valley University, Qena, 83523, Egypt

**Keywords:** Arsenic, Bioremediation, Biotechnology, Contaminant removal, Genome

## Abstract

Heavy metal contamination threatens the aquatic environment and human health. Different physical and chemical procedures have been adopted in many regions; however, their adoption is usually limited since they take longer time, are more expensive, and are ineffective in polluted areas with high heavy metal contents. Thus, biological remediation is considered a suitable applicable method for treating contaminates due to its aquatic-friendly features. Bacteria possess an active metabolism that enables them to thrive and develop in highly contaminated water bodies with arsenic (As). They achieve this by utilizing their genetic structure to selectively target As and deactivate its toxic influences. Therefore, this review extensively inspects the bacterial reactions and interactions with As. In addition, this literature demonstrated the potential of certain genetically engineered bacterial strains to upregulate the expression and activity of specific genes associated with As detoxification. The As resistant mechanisms in bacteria exhibit significant variation depending on the genetics and type of the bacterium, which is strongly affected by the physical water criteria of their surrounding aquatic environment. Moreover, this literature has attempted to establish scientific connections between existing knowledge and suggested sustainable methods for removing As from aquatic bodies by utilizing genetically engineered bacterial strains. We shall outline the primary techniques employed by bacteria to bioremediate As from aquatic environments. Additionally, we will define the primary obstacles that face the wide application of genetically modified bacterial strains for As bioremediation in open water bodies. This review can serve as a target for future studies aiming to implement real-time bioremediation techniques. In addition, potential synergies between the bioremediation technology and other techniques are suggested, which can be employed for As bioremediation.

## 1 Introduction

Heavy metal contamination threatens the aquatic environment and human health [1,2]. Numerous heavy metals such as chromium (Cr), lead (Pb), zinc (Zn), arsenic (As), copper (Cu), cadmium (Cd), nickel (Ni) and mercury (Hg) are the main cause of the contamination of surface and/or groundwater [3]. The aquatic environment might be polluted through septic systems, waste from storage tanks, landfills, hazardous waste sites, pesticides and the application of fertilizers [4,5]. It is recognized that arsenic (As) is a particularly hazardous element in aquatic environments, particularly in its inorganic chemical structure form [6]. The oxidation of sulfide-rich arsenopyrite rocks results in As-containing acid mine drainage, which is a major by-product of the mining of coal, with a total As content exceeding 20 mg L^−1^ [7]. The discharge of arsenopyrite into the aquatic environment generates two primary forms of As (trivalent arsenite, As III, and pentavalent arsenite, As V) [8]. Specifically, As III is the major chemical form of arsenic pollutants of drainage from acid mines with severe environmental consequences (such as, weathering of bedrock, arsenopyrite oxidation and chief sulfides in rocks, the mine water and surface water mixture, leaching of the arsenic alkali residue, and adsorption-desorption from iron/manganese oxide/hydroxide), and it might be changed into a less hazardous form (As V) in an aerobic condition [9]. As III has a notable negative impact on aquatic organisms due to its weak binding to mineral surfaces. The weak binding is a result of As III being electrically neutral, unlike the negatively charged As (V) oxyanions [10]. Meanwhile, acute or chronic As exposures cause negative health consequences such as cardiovascular disorders, cancer threats, or even sudden death at lethal concentrations [11]. Therefore, the World Health Organization (WHO) has recognized a high permissible level of 10 μg/L for As in drinking water [12]. A recent study discovered that 61 out of 64 districts have higher arsenic levels in their drinking water that exceed the recommended limit set by the World Health Organization (10 μg/L) [13]. This is a significant issue affecting over 85 million people. It is worth noting that shallow groundwater in sedimentary lacustrine aquifers tends to have higher arsenic levels, whereas deeper aquifers (>300 m) are generally considered safe [14]. Thus, there is a rising interest towards detecting the hazardous effects of arsenic on aquatic species and its indirect consequences on human health [15], as well as finding novel innovations to reduce its toxicity.

Conventional physical adsorption, membrane separation or ion exchange, oxidation or coagulation using chemical substances, precipitation, and phytoremediation methods remove As from contaminated water [16], but microbial-based As bioremediation techniques are cutting-edge and cost-effective. Microorganisms are essential in the process of bioremediation because they can break down complex organic compounds into simpler substances [17]. The application of microorganisms in bioremediation is a captivating subject that has been extensively researched by scientists from various fields, such as microbiology, environmental science, and biotechnology [18]. Specific microbial strains have the potential to break down As compounds into less harmful forms and may live in polluted sites because of their metabolic features [11]. Several investigations proved the efficiency of Arsenite oxidation employing *Thermus* species [19], methylation of arsenite using genetically modified *Pseudomonas putida* [20] and As adsorption via *Escherichia coli* [21] as well as converting its toxic form (As III) into low bio-accumulative and toxic chemical forms. It has been previously verified that genetically engineered bacterial strains had greater As bioaccumulation efficiency than natural bacterial strains [22]. Thus, it is vital to investigate genetic factors that have highly successful As adsorption capability and susceptibility in heterologous hosts. Moreover, the use of genetically engineered bacterial strains in bioremediation offers numerous advantages. These include low costs, eco-friendly properties, and a socially acceptable approach for the sustainable elimination of heavy metal waste [23]. However, genetically engineered microbes for bioremediation have not been extensively utilized in the treatment of natural water bodies or aquaculture farms. The main reason for this is that they are generally not permitted to be released into the surrounding waters [[24], [25], [26]]. In particular, the application of microorganisms in bioremediation has various obstacles, such as the high danger of creating secondary chemicals and the discharge of modified genes into natural habitats, which must be resolved before adoption [27]. Subsequently, researchers might ensure the secure application of genetically engineered microbes for As bioremediation using technological protections and suitable approval regulations, such as accomplishing thorough risk assessments and monitoring [28]. Accordingly, bioremediation is a research-critical approach that requires detailed knowledge of microbial modes of action [29].

As mentioned above, numerous studies have thoroughly examined the various forms of arsenic and the role of genetically modified bacterial strains in remedying arsenic contamination in different environments [18,30]. However, there is still a lack of understanding regarding As contamination, discharge into aquatic bodies, bioaccumulation, and bioremediation mechanisms specifically in aquatic systems. With this in mind, the present review aims to achieve two objectives: (1) provide a concise overview of As discharge and contamination in aquatic environments, as well as the application of biotechnology in water treatments, and (2) discuss the potential benefits of utilizing gene encoding techniques to create genetically modified bacterial strains that are highly effective in bioremediation. Furthermore, we will analyze the main challenges that arise when utilizing genetically modified organisms (GMOs) in water treatment, focusing specifically on marine and freshwater environments.

## 2 Discharge of arsenic into aquatic bodies

Arsenic arises via mother natural mechanisms, including erosion of lava flows, stones containing As and some other biological and human activities, such as operations related to mining, electroplating, metal smelting and application of synthetic fertilizer and/or pesticide in aquatic areas [31]. According to earlier reports, As levels in surface and groundwater near smelting and sulfide mining locations might fluctuate from 100 to 5000 μg/L [32]. Meanwhile, larger quantities of As (1386 up to 5850 μg/L) have been detected offshore near hydrothermal systems [33]. Thus, arsenic pollution of aquatic environments is considered a serious ecological hazard, impacting over 115 countries worldwide [34].

In surface water regions, arsenic is converted into complex chemical compounds. Specifically, there are two main forms of inorganic As types consist of arsenate (As V) and arsenite (As III) [35]. In addition, there are many complicated forms of organic As types such as, dimethylarsinate (DMA), methylarsonate (MA), trimethylarsine oxide (TMAO), tetramethylarsine (TMA), arsenobetaine (AsB), arsenocholine (AsC), arsenosugars (As-Sug), thiolated arsenic, and arsenolipids [36]. After releasing As into water bodies, it has an enormous adverse impact on aquatic species (Table 1), either directly via drinking and breathing or indirectly through increased absorption levels throughout the natural food chain [37]. Additionally, arsenic may threaten consumer health by contaminating drinking water or accumulating into aquatic by-products [38]. Worldwide, from 94 to 220 million individuals, with the vast majority (94 %) living in Asian nations, may be vulnerable to very high amounts of As accumulating in groundwater [39]. According to the US Food and Drug Administration, human consumers may acquire 90 % of the total As by consuming marine fish and other seafood products [40]. Thus, knowing both the biological and ecological pathways of As is very important.

**Table 1:** The adverse effects of arsenic contaminated water bodies on aquatic creatures.

Arsenic forum	Aquatic species	level	Duration	Biological effect	References
Arsenic and inorganic arsenic	macroalgae *species L*. *digitata* and *S*. *latissima*	(41 mg kg−1 and 43 mg kg−1, respectively)	Chronic	Oxidative stress, metabolic disturbances, and histological alterations in the gills and liver.	[41]
As(V)	Marine juvenile fish *Terapon jarbua*	50 μg/L	2, 4, 6, and 8 h	The marine fish primarily converted the accumulated As(V) into the non-toxic organic arsenic compound known as arsenobetaine (AsB).	[42]
Arsenobetaine and arsenate	The marine grouper (*E. fuscoguttatus*)	500 mL of freshly prepared solutions of AsB (500 μg of AsB added as C5H11AsO2) and As(V) [500 μg of As(V) added as Na2HAsO4·7H2O] were added to 500 g of unmodified food pellets, respectively.	14 days	AsB exhibited a diminished capacity for passing through the intestinal membranes, resulting in sluggish absorption and eventual storage in muscle. In contrast, As(V) demonstrated rapid crossing of the intestinal membranes, swift transportation, and elimination.	[43]
Inorganic As species (As(III) and As(V))	Marine herbivorous fish *Siganus fuscescens*	400 and 1500 μg As(III) or As(V) g^−1^ (dry weight)	21 days and 42 days	It was showed that both inorganic forms of arsenic, As(III) and As(V), present in the diet, were capable of undergoing biotransformation into the less harmful compound arsenobetaine (AsB). This biotransformation occurred to a range of 63.3 %–91.3 % in the liver and 79.0 %–95.2 % in the muscle.	[44]
Arsenate (As(V)) and arsenite (As(III)	Freshwater fish crucian carp (*Carassius auratus*)	50 and 100 μg As(III) or As(V) g^−1^ (dry weight)	10 days and 20 days	The conversion of As(V) to As(III) and the conversion of As(III) to As(V) took place in fish that were fed with As(V) and As(III) respectively.	[45]

Research has demonstrated that the concentration of different forms of arsenic (As) in freshwater ecological systems is influenced by the process of biodilution [46]. However, in marine habitats, the concentration is more closely related to the enrichment of organic forms, specifically arseno-betaine. Consequently, this leads to the deterioration of food containing high levels of arseno-sugar [47]. Particularly, AsB is regarded as the outcome of As degradation in the marine live food cycle, although mechanistic knowledge of its biodegradation and production remains limited [48]. Several variables influence arsenic bio-absorption by aquatic creatures, including aquatic species, organism weight and age, as well as some ecological aspects such as salinity, pH, phosphorus, and dissolved organic matter (DOM) concentrations [35]. In addition, there are some factors related to the nature of the aquatic organism that affect arsenic transformation into food chain including the rate of ingestion, the gut's entire environment, the gut passage time, aquatic species, the consuming rate of living prey, stocking density, pH value, and iron oxides concentration [49]. Furthermore, various physiological activities including reproduction, excretion, and molting contribute to the body's elimination of As [50]. Besides, the density of live feed, pre-exposures, prey types, and phosphate level might influence the As elimination efficiency [51]. Thus, it is vital to highlight the available knowledge on the bioaccumulation of As to generate a single, unified resource for future research.

## 3 Mechanisms of bacterial bioremediation for arsenic

**Fig. 1:**
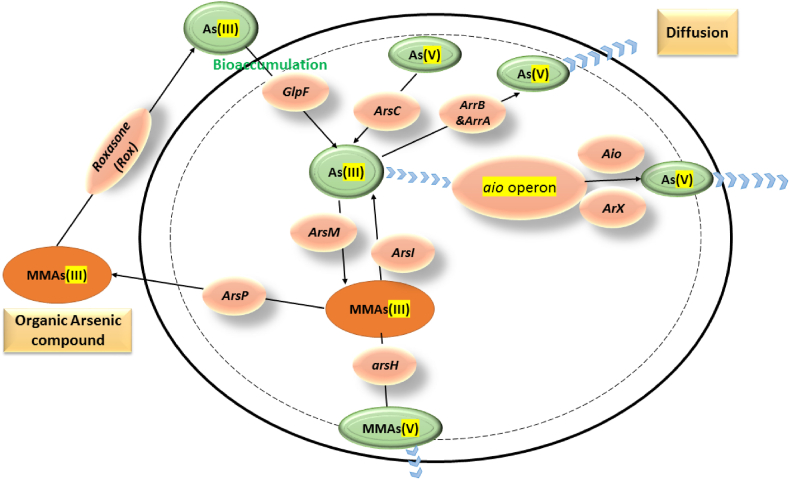
Summarizes the genetic strategies used by genetically modified bacterial cells to identify biotransformation, reduction, and oxidation pathways in aquatic environments contaminated with arsenic. These strategies include the use of specific genes and operons.

Bacteria are very resistant to difficult environmental conditions and might live by maintaining varied biological processes, allowing them to thrive and grow in severe habitats [52]. Multiple bacterial strains have the potential to grow well in arsenic-contaminated environments and exhibit diverse arsenic detoxification pathways via multiple methods that allow them to avoid the harmful effects caused by arsenic [53]. The list of some bacterial stains applied for arsenic remediation is summarized in Table 2 content. Several reports demonstrated several bacterial detoxification processes against arsenic pollution, including the production of exopolysaccharide (EPS), complexation via specific proteins, reduction or oxidation reactions, precipitation, methylation process, metal chelating, cellular surface adsorption or biosorption mechanisms, metal entrapment by cellular capsulation, and active biological transportation [54,55]. These detoxification pathways of arsenic may be applied for bioremediation of arsenic. In order to effectively remove arsenic from aquatic wastewater using gene-transforming bacterial strains, a deep understanding of all the processes involved in this method is essential. Therefore, the following are the arsenic bioremediation methods identified in various bacteria (Fig. 1), allowing microorganisms to be used in wastewater treatment applications.

**Table 2:** The list of bacterial stains applied for arsenic remediation.

Bacterial strain	Arsenic level (mg/L)	Arsenic form	Bioremediation mechanisms	Remediation rate (%)	Maximum pH value	Incubation maximum Temperature degree	References
*Exiguobacterium profundum*	71,924.73 or 600	As^5+^ or As^3+^	Biosorption	N/A	7	37	[56]
*Bacillus aryabhatti*	37,500	As^5+^	Reduction	N/A	7	60	[57]
*Micrococcus* sp.	44,953	As^5+^	Oxidation	N/A	9	30	[58]
*Bacillus flexus*	11,239 or 5245	As^5+^ or As^3+^	Biosorption	8	8	30	[59]
*Acineto bacterjunii*	11,239 or 5245	As^5+^ or As^3+^	Bioaccumulation	14	8	30	[60]
*Bacillus indicus*	22,477 or 150	As^5+^ or As^3+^	Bioaccumulation	N/A	7	37	[61]
*Acineto bacterlwoffii*	26,223 or 375	As^5+^ or As^3+^	Bioaccumulation	N/A	7	30	[62]
*Acinetobacter* sp.	29,969 or 375	As^5+^ or As^3+^	Bioaccumulation	N/A	9	30	[62]
*Acinetobacter* sp.	26,223 or 1124	As^5+^ or As^3+^	oxidation	N/A	7	37	[63]
*Exiguobacterium* sp.	26,223 or 750	As^5+^ or As^3+^	Oxidation and reduction	N/A	7	30	[64]
*Bacillus* spp.	74,922 or 5245	As^5+^ or As^3+^	Oxidation and reduction	88.74	10	30	[65]
*Pseudomonas* sp.	974	As^5+^	Oxidation	100	7	30	[66]
*Pseudomonas* sp.	1124	As^3+^	reduction	100	7	30	[67]
*Providenciarettgeri*	1000	As^5+^	Bioremediation	N/A	8.5	35	[68]
*Pseudomonas chengduensis*	20280 or 3250	As^5+^ or As^3+^	reduction	48–78	6	37	[69]
*Acinetobacterl woffii*	9365 or 3746	As^5+^ or As^3+^	Bioremediation	N/A	7	30	[59]
*Leclerciaadecarboxylata*	7492 or 750	As^5+^ or As^3+^	reduction	100	7.5	37	[66]
*Pseudomonas aeruginosa*	7000 or 1400	As^5+^ or As^3+^	Biosorption	98	7	30	[70]
*Bacillus cereus*	3000	N/A	Bioaccumulation	83.81	7	30	[71]
*Lysinibacillusbor Onitolerans*	3000	N/A	Bioaccumulation	85.72	7.5	30	[72]
*Delftia* spp.	74,922 or 5245	As^5+^ or As^3+^	oxidation	91.04	7	37	[70]
*Micrococcus* sp.	29,969 or 1873	As^5+^ or As^3+^	oxidation	39.22	7	30	[73]

### 3.1 Detoxification supported by an active biological transportation activity

The protein transporter generated via the *arsP* gene can carry the organic form of arsenic directly inside the bacterial cells. Specifically, the *arsN1*, *arsI,* and *arsH* genes generate specific enzymes that participate in organoarsenical detoxification [74]. Few types of bacteria contain *arsI* gene that is responsible for generating the lyase enzyme, which is capable of breaking down carbon-arsenic bonds and altering organo-arsenicals to Arsenic III [75]. Whereas, the *arsH* gene could oxidize trivalent aromatic arsenicals and methylated via the organo-arsenical oxidase enzyme and transform them into pentavalent structure form [76].

On the other hand, certain soil bacterial strains convert organo-arsenical to the very poisonous pentavalent form known as arsinothricin [77]. Meanwhile, bacteria developed in arsinothricin-contaminated ecosystems may detoxify arsinothricin compounds by converting α-amino groups to methyl groups through acetylation process [78]. Recent research has shown that *E. coli* strains include another type of *arsN1* gene (PparsN1), which has the potential to convert arsinothricin into a less hazardous form by activation of the acetylation pathways process [79,80].

In addition, bacteria have an operon system, which is a genetic regulatory system that arranges genes encoding similar proteins together with the DNA. This operon system is used by bacteria as a mechanism for bioremediation of heavy metals [81]. In the presence of heavy metals, the regulatory protein associated with the promoter region interacts with the metals, enabling DNA polymerase to bind to the promoter. As a result of this interaction, various systems within the operon, such as efflux systems, conversion systems, and multiple types of resistance systems, are transcribed [82].

Meanwhile, some microbes that are resistant to arsenic can use As V in anaerobic respiration or convert As V to As III as a way to detoxify [83]. The features of these strains of bacteria, which have resistance mechanisms, are encoded by the *ars* operon. The configuration of the operon varies among different strains [84]. The regulatory protein ArsR, which stimulates the specific binding site for As III; the As V reductase ArsC; and the As III efflux pump ArsB [83]. Whereas, ArsC facilitates the reduction of As V using glutaredoxin, glutathione, or thioredoxin. This detoxification pathway requires ATP as an energy source [85].

In addition, the *arr* operon consists of two genes, *arrA* and *arrB*, which encode the large and small subunits of *Arr*, respectively. Therefore, *Arr* is a functional heterodimeric periplasmic protein that requires the expression of both *ArrA* and *ArrB* subunits. In bacterial strain ANA-3, *Arr* expression begins during the exponential growth phase and continues throughout the stationary phase until it is released from the cell [86]. The protein's activity is not activated by electron acceptors such as antimonite, nitrate, selenate, and sulfur [83]. However, recent research has shown that *ArrA* is specifically induced in the presence of As(V) and acetate in *G. lovleyi* [87], highlighting the role of these bacteria in releasing arsenic from groundwater sediments.

A recently discovered *arx* operon, similar to MLHE-1, has been found in the genome of *Ectothiorhodospira* sp. strain PHS-1 [88]. This bacterium, which is a purple sulfur bacterium, carries out photosynthesis and was isolated from Mono Lake (hydrothermal waters). It can utilize As(III) as an electron donor in anaerobic phototrophy [89]. Another strain, called ML-SRAO and also isolated from Mono Lake, can anaerobically oxidize As (III) and reduce selenite [90]. Unlike MLHE-1, ML-SRAO cannot grow autotrophically, but it can develop heterotrophically on lactate using As V as the electron acceptor. The absence of As III oxidase gene amplification and the positive amplification of the *arrA* gene from strain ML-SRAO indicate that *arrA*, like MLHE-1, functions as an oxidoreductase [83].

Finally, resistant bacterial strains have an *aio* operon in their genome structure, which is responsible for the oxidation pathways of As (III) [91]. In the same context, Hao et al. [92] reported the presence of the *aio* operon involved in As (III) oxidation within the genome of *A. tumefaciens* 5A (As (III)-oxidizing strain). The expression of this operon is regulated by a two-component signal transduction system and quorum sensing [92].

### 3.2 Biosorption pathways

Biosorption is an effective method used for eliminating pollutants from contaminated environments [93]. Microorganisms can biosorb arsenic because of their more advanced cell wall architecture [59]. Because of the abundance of arsenic-binding regions on the bacterial cell wall, arsenic biosorption increased notably throughout the early stages of bacterial growth. The biosorption of arsenic decreased in late bacterial developmental stages owing to increasing the saturation of heavy metal sites of binding [56]. Bacterial fluids produced in reaction to As assist aggregate As from the environment onto its cell membrane [56].

The biosorption of As relies on the binding of As containing a positive charge with the bacterial cell wall or extracellular fluids having a negative charge [94]. Besides, some function groups remain within the bacterial cell wall actively biosorbent Arsenic III, such as carboxyl, hydroxyl, amine, and amide [95]. Furthermore, the bacterial cell wall biofilm may operate to serve as an effective biosorbent agent for the As owing to the presence of a high quantity of exopolysaccharides [56]. For instance, *Exiguobacterium profundum* demonstrated elevated As biosorption efficiency in both planktonic and biofilm forms [56].

There are several factors have been influencing the bacterial biosorption efficiency of As such as ecological factors (for instance, pH, temperature, *etc*.), the bacterial biomass chemical structure and solution physicochemical features [93]. It was investigated that Arsenic V biosorption rate significantly reduced with higher ambient temperature [96]. The influence of ambient temperature on biosorption effectiveness might be attributed to the diminishing of physical properties and instability of the surface of the adsorbent at higher degrees [96]. Meanwhile, the solution's pH directly impacts arsenic biosorption by altering the bacterial functional groups and the arsenic form [96]. In alkaline solution, the As biosorption significantly decreased with higher pH values. While, bacterial cell wall exhibits high As ш biosorption in acidic environments between pH 4–7 with increasing the bacterial negative charge to bind As ш positive charge [93]. Thus, controlling environmental variables might enhance the biosorption rate for As remediation.

Several bacterial strains have shown the biosorption efficiency of arsenic. Isolated bacterial stains from groundwater, such as *Pseudomonas aeruginosa*, demonstrated higher biosorption efficiency (90.72 %) towards arsenic, decreasing it from 10,000 down to 928 ppb in 30 min. Also, increasing incubation time enlarged biosorption efficiency (97.92 %) down to 208 ppb in 2 h [67]. Moreover, E. *Profundum* is exhibited to have As biosorption capability [56]. In addition, fermented bio-waste by *Corynebacterium glutamicum* covered with polyethylenimine has shown higher biosorption efficacy, removing 62.99 mg per g of As V [96]. Meanwhile, employing *Yersinia* sp. dead cells showed higher biosorption efficiency (47 %) of Arsenic III in 80 min under an acidic environment [97].

### 3.3 Bioaccumulation

Bioaccumulation in bacteria is a metabolic mechanism that requires energy to absorb heavy metals within the cell and accumulate them in its cytoplasm [59]. Bacteria can transfer arsenic from polluted areas into their cells by a variety of methods, including exchange of ions, medium transport, channels of ionization, endocytosis, and fat permeability [[98], [99], [100]]. Besides, Arsenic might be retained within the cellular cytoplasm in a variety of forms, including meta-arsenite, free arsenic, arsenate, and ortho-arsenite [101]. These arsenic types might be connected to entire cellular biomolecules or retained in lipid vacuoles to eliminate their cellular negative effects [102]. Recently, it was explored that deactivating certain enzymes, such as arsenate reductase and modifying the bacterial cell efflux routes (*ACR3*) enhanced arsenic bioaccumulation within the cell [103]. For instance, the absence of *aCR3* and arsenate reductase improved *C. glutamicum*'s arsenic bioaccumulation capability by 28–30 times [98]. Furthermore, the particular protein transporter upregulation (GlpF) enhances arsenic bioremediation, subsequently increasing the bacterial bioaccumulation performance [101]. Moreover, bacterial stains developed in an arsenic-contaminated aquatic solution with 100 ppm of Arsenic V or Arsenic III showed higher bioaccumulation capacity throughout the log phase of development [7]. Whereas, *Pseudomonas* and *Exiguobacterium* showed higher arsenic bioaccumulation efficiency up to 17.58 and 19.66 mg per g during 24 h, respectively [104]. Thus, microorganisms having a high potential for arsenic bioaccumulation may be beneficial for arsenic bioremediation in polluted locations.

## 4 Biotechnology's role in arsenic bioremediation

Numerous biotechnological methodologies characterize the efficacy of identified molecular impact on As remediation [7]. In recent decades, scientists have produced genetically altered microbes that are resistant to and accumulate arsenic. The main microbial genetically modified strains applied in arsenic bioremediation were summarized in Table 3 content. For instance, it had been previously demonstrated that the *Bacillus subtilis* 168 strains modified genetically using the upregulation of the CmarsM gene (arsenite S-adenosylmethionine methyltransferase) from the alga *Cyanidioschyzon merolae* resistance to overheating showed a higher ability to volatilize and methylate arsenic [73]. Heterologous upregulation of ArsM from *Rhodopseudomonas palustris* conferred As III tolerance to an arsenic-sensitive strain of *E. coli*. While, ArsM catalysis is the synthesis of a variety of methylated intermediates from As III, resulting in trimethylarsine. The end result is the removal of As from both the medium and the cells [105]. It was found that the arsenic M gene was markedly upregulated in *Bacillus idriensis* and *Sphingomonas desiccabilis* [106]. Furthermore, it was demonstrated that genetically modified microorganisms with highly upregulated arsenic M gene can accumulate Arsenic (for instance, from 2.2 up to 4.5 % in the soil and more than 10-fold in aquatic solution nutrients) via bio-volatilization compared to the natural bacterial strains [28]. Meanwhile, modified *Bacillus subtilis* 168 strains through overexpression of the CmarsM gene turned the arsenic into its methylated form (trimethylarsine and dimethylarsenate oxide) or converted it into volatilized form (trimethylarsine and dimethylarsine) [55]. Also, when the genetically engineered bacterial strains were put into arsenic-polluted organic waste for 48 h, they transformed inorganic arsenic into its volatilization form and produced methylated organic molecules [54]. All of the above-mentioned investigations evaluated the successful function of using genetically modified microbes for bioremediation of Arsenic-polluted compost. The genetically engineered *Corynebacterium glutamicum* stain resistance to arsenic demonstrated optimized bioremediation removal efficiency of arsenic [107]. Specifically, the genetically engineered *Corynebacterium glutamicum* stain converted arsenic via mycothiol-based single-cysteine reductase into two major forms (Arsenic C1 and Arsenic C2) [72]. Also, the genetically modified *Corynebacterium glutamicum* strain has the potential to create three-cysteine homodimer Arsenic C1, boosted by the arsenic 1 operon, which has a constitutive function to boost Arsenic (V) absorption in this strain [108]. Thus, the genetically engineered *Corynebacterium glutamicum* strains were capable to bioremediate 30-fold more arsenic (V) and 15-fold more arsenic (lll) compared to the untreated group [109].

**Table 3:** The main microbial genetically modified strains applied in arsenic bioremediation.

Strain	Family	Bioaccumulation efficiency	Expressed genes	References
*Desulfovibirio desulfuricans*	Desulfovibrionaceae	Biomineralizes pyrite for arsenic removal	Not identified	[110]
*Paraclostridium* sp. EML	Clostridiaceae	This bacterium is capable of methylating arsenic under anaerobic circumstances and effectively detoxifying the resulting compound.	*arsM*	[111]
*Pseudomonas* (AK1 and AK9)	Pseudomonadaceae	The presence of aoxR, aoxB, and aoxC genes in both isolated strains AK1 and AK9 has been established. These genes are crucial in the process of arsenic bioremediation through the oxidation of arsenite.	*aoxR*, *aoxB* and a*oxC*	[112]
*Pseudomonas alcaligenes*	Pseudomonadaceae	This strain has the capability to methylate and volatilize arsenite.	*arsB*	[44]
KG1D and PF14	Pseudomonadaceae	The organism undergoes oxidation of hazardous arsenic, exhibits tolerance towards several other heavy metals, and effectively eliminates arsenic.	*arsRBC*	[95]
*Rhodococcus* sp. TS1, *Delftia* sp. TS33, *Delftia* sp. TS41, *Streptomyces lividans* sp. PSQ22 and *Comamonas* sp. TS37	Nocardiaceae, Comamonadaceae, and Streptomycetaceae	Arsenate-reducing mechanism	Not identified	[113]
*Brevibacterium linens* strain AE038-8	Brevibacteriaceae	Extremely arsenic-resistant and can reduce As V to As III in specific conditions.	*aCR3*, *arsC*, *arsR* and two isolated types of *arsO*	[114]
*Achromobacter xylosoxidans* BHW-15	Alcaligenaceae	This strain has the ability bioaccumulate and detoxify trivalent arsenic.	*aioA*	[115]

Meanwhile, it was previously observed that amplification of the arsenic R gene in bacterial strains with elastin-like polypeptide (ELP153AR) boosted arsenic bioaccumulation throughout their cells 60-fold compared to non-modified bacterial cells [116]. Genetically modified *E. coli* cells with higher upregulation of the arsR gene optimized the effectiveness of its bioremediation removal properties up to 100 % of the arsenite (50 ppb) in the polluted site [104], indicating that genetically modified organisms indicate a less expensive and more efficient technique for future applications in bioremediation of arsenic. In *Pseudomonas putida* strain KT2440, the overexpression of arsR1 and arsR2, the primary two arsenic-sensitive inhibitors, indicated their function as regulatory triggers in arsenite and arsenate resistance [20]. In vitro analysis of the aforementioned genes showed that one regulator form (arsR1 or arsR2) inhibits the other [117]. The present investigation proposes that the presence of Arsenic R gene mutations in the bacterial cells influences their adaptive role as suppressor genes of arsenic resistance [118].

## 5 Applying gene encoding techniques to produce genetically modified bacterial strains

Several genetic engineering methods at the gene or genome level have been employed to insert, remove, or substitute one or more nucleotides into bacterial DNA fragments to generate genetically engineered bacterial strains. Recently, CRISPR-Cas (clustered regularly interspaced short palindromic repeats-associated nucleases) techniques have been developed to be an effective and simple gene editing approach [119]. Meanwhile, certain DNA-binding application techniques have been implemented at the entire genome level, such as TALEN (transcription activators like effector nucleases), which is nucleotide sequences particular to the host genome [120]. While, the DSBs (Double-stranded breaks) approaches were developed for stabilizing the DNA fragments produced by the TALEN process [121]. Additionally, the ZFNs (Zinc-finger nucleases) method was used in conjunction with the presence of 30 amino acids to increase DNA bending dominance [122]. Furthermore, the *Fok*I DNA cleavage domain has played a pivotal role in the success of ZFNs and later TALENs [123].

Therefore, two major approaches (TALENs and ZFNs) have arisen in parallel to cope with the molecular complications of nucleases [118]. Consequently, the technique known as CRISPR-Cas is capable of editing multiple genes rapidly with a high precision degree [23]. Recently, several attempts have been made to apply CRISPR-Cas tools in microorganisms like *E. coli* or *Pseudomonas*, or even non-model bacterium strains like *Comamonas testosteroni* and *Achromobacter* sp. HZ01 to determine its bioremediation effectiveness in arsenic bioremediation via verifying the generation of gRNA that encodes function-altered specific genes relevant to the remediation process [104]. Specifically, CRISPR-Cas9 could be employed to delete or insert the specific gene of interest in the bacterial strain to stimulate its bioremediation efficiency [124]. For example, removing the yvmC gene from *Bacillus licheniformis* cell via the CRISPR-Cas9 technique, raising its bioremediation efficiency to 100 % [125]. Thus, CRISPR-Cas9 technology could be employed soon to create new strains of microbes capable of bioremediating arsenic in the aquatic environment.

Besides, metabolic engineering techniques could enhance the bioremediation efficiency of bacterial strains by inserting specific genes responsible for up-regulating the synthesis of particular enzymes such as esterases, oxidases, phenoloxidases, monooxygenases, and oxidoreductases that are responsible for a bacterial enzyme-based bioremediation method (called green method) [59]. As mentioned above, the arsenic detoxifying pathway is regulated by ArsC-encoding genes, which stimulate proteins that promote the reduction of Arsenic V into Arsenic III using arsenate reductase enzyme in the cytoplasm [126], followed by Arsenic decontaminate removal from the cytoplasm by an outflow channel [19]. Thus, the availability of metabolic pathway information is vital in determining the microbial bioremediation efficiency of heavy metals.

## 6 The awareness regarding the wide application of genetically altered bacteria in arsenic bioremediation

There are many issues and restrictions with using genetically modified bacteria for arsenic bioremediation that hinder its widespread use in ecological areas. Major drawbacks or restrictions with bioremediation applications using engineered bacterial strains include (1) the possibility of creating secondary metabolites and leakage of genetically modified genes into aquatic systems, which must still be disclosed before widespread commercial use. Researchers compared the maximum documented levels of accumulation of arsenic in the published literature and discovered that wild live bacteria could accumulate 9.80 ± 0.50 mg As/g dry-weight bacteria [71], but engineered strains could accumulate 7.59 mg As/g dry-weight bacteria [127]. Therefore, it is essential to determine the genetic factors that have a high sensitivity and ability to stimulate arsenic adsorption in heterogeneous hosts [111]. Also, (2) it is important to carefully analyze the different distribution of contaminants in treated areas since this might lead to an unbalanced scattering of the gas or liquid generated by applied modified bacteria. Because acceptable levels of decontamination are being achieved at different sites at a slower rate, it is difficult to predict with certainty how biological remediation will be employed widely in open areas with expected success [28]. Furthermore, (3) the release of genetically engineered bacterial stains into open areas is a complex procedure that has to adhere to strict guidelines and get authorization from several ethical and regulatory organizations [95]. Thus, utilizing genetically engineered bacterial strains for bioremediation is a cutting-edge technique that involves extensive knowledge of the metabolic pathways of the microbial population.

## 7 Conclusion and future directions

Arsenic (As) is a heavy metal metallic ion found in aquatic systems as a consequence of both natural (volcanic eruptions, fires in forests, and rock erosion) and human activities (including the wastes of paint, medication, herbicide, shampoo, and electronics industries). It is a substantial and widespread environmental pollutant that has direct and indirect health consequences on all living creatures. Thus, complete or partial elimination of this heavy metal from water bodies is a critical process for ecological remediation in many regions. There are various technologies suggested for removing arsenic from aquatic environments, including precipitation using chemicals, exchange of ions, adsorption, filtration through membranes, botanical remediation, bacterial bioremediation, and electrocoagulation. Several recent reports have proven that the efficacy of employing bacterial strains for arsenic bioremediation of contaminated water improved significantly after modifying the genetic structure of these bacteria. Specifically, genetically engineered bacterial strains can boost the quantity of released enzymes that can break down both inorganic and organic forms of arsenic in the aquatic environment. Until now, additional investigations were required to identify the specific bacterial strains that have a high ability to break down inorganic arsenic contaminants.

The water quality criteria (such as, pH and ambient temperature fluctuation) are the major issue that should be thoroughly examined to maintain high bacterial bioremediation efficiency. In addition, further studies are required to determine the ideal ambient temperature and other water physical requirements for the breakdown of arsenic molecules in natural water bodies. Furthermore, understanding the relationship between the water ambient temperature degree and the incubation temperature necessary for bacterial growth to survive in the aquatic environment is critical. This could help prevent the failure of arsenic bioremediation when releasing or mobilizing exogenous modified bacterial strains into an aquatic environment.

Thus, it is necessary to undertake more research to prove the safety of employing genetically modified organisms in wastewater treatment by evaluating all environmental implications that may occur via mutation and other processes. Therefore, regulatory agencies should be established to monitor any potential problems associated with genetically altered bacterial species, especially in aquatic environments, as well as encourage lawmakers and public people to support this bioremediation technique.

## Ethical approval

Not applicable for review studies.

## Funding

Not applicable.

## Consent to participate

Not applicable.

## Consent to publish

Not applicable.

## Data availability statement

The collected literature applied and/or presented during the current study is available from the corresponding author (Mohammed A.E. Naiel) on reasonable request.

## CRediT authorship contribution statement

**Mohammed A.E. Naiel:** Writing – review & editing, Writing – original draft, Formal analysis, Data curation, Conceptualization. **Ehab S. Taher:** Funding acquisition, Formal analysis. **Fatema Rashed:** Investigation, Funding acquisition. **Shakira Ghazanfar:** Funding acquisition, Formal analysis. **Abdelrazeq M. Shehata:** Validation, Software. **Nourelhuda A. Mohammed:** Validation, Supervision. **Raul Pascalau:** Methodology, Formal analysis. **Laura Smuleac:** Visualization, Validation. **Ateya Megahed Ibrahim:** Software, Resources, Project administration. **Ahmed Abdeen:** Writing – original draft, Supervision, Software, Investigation. **Mustafa Shukry:** Visualization, Resources, Methodology.

## Declaration of competing interest

The authors declare that they have no known competing financial interests or personal relationships that could have appeared to influence the work reported in this paper.
